# Integrating Community Care for the Prevention and Treatment of Diabetes

**DOI:** 10.5334/ijic.7607

**Published:** 2024-06-04

**Authors:** Katsuya Fuse, Norihito Kamimura, Seitaro Iguchi, Kiminori Kato, Hideaki E. Takahashi

**Affiliations:** 1 Uonuma City Koide Hospital, 34-34 Hiwatari-shinnden, Uonuma-shi, Niigata-ken, 946-0001, Japan; 2 Kamimura Clinic, 12 Suwamachi 1-chome, Uonuma-shi, Niigata-ken, 946-0003, Japan; 3 Department of Community Medicine, Niigata University Graduate School of Medical and Dental Sciences, 1-757 Asahimachi-dori, Chuoh-ku, Niigata-shi, Niigata-ken, 951-8122, Japan; 4 Department of Prevention of Noncommunicable, Diseases and Promotion of Health Checkup, Niigata University Graduate School of Medical and Dental Sciences, 1-757 Asahimachi-dori, Chuoh-ku, Niigata-ken, 951-8522, Japan; 5 Niigata University, Co-Chair, Niigata Initiative for Promotion of Health and Welfare, Japan; 6 Niigata Rehabilitation Hospital, Niigata Bone Science Institute, 761 Kizaki, Kita-ku, Niigata-shi, 950-3304, Japan

**Keywords:** integrating community care, Project 8, HbA1c, interprofessional education, interprofessional collaborative practice, community resident participation

## Abstract

**Introduction:**

This paper describes ‘Project 8’, a campaign that aims to reduce glycated haemoglobin (HbA1c) to 8% or more among patients with diabetes mellitus, utilising healthcare professionals and local community residents and focusing on education and support. The study is based in Uonuma—a small rural city in Japan with a declining population and an increased number of older people.

**Description:**

‘Project 8’ began in Uonuma’s Koide Hospital in 2008. The Uonuma School for Community Health and Social Care was established in 2011 with the cooperation of a clinic’s general practitioner. Medical students, trainees, doctors, and health care professionals have been holding ‘open schools’ (daytime lectures) and ‘night schools’ (evening lectures) to educate the community residents about various health issues. Through repeated lectures, the residents have been made aware of lifestyle-related diseases, including diabetes, and the meaning of ‘Project 8’.

**Discussion:**

Over the last decade, the hospital’s campaign has expanded within the community, showing a statistically significant reduction of diabetic patients with HbA1c ≥ 8%, which successfully deferred the start of dialysis for many of them.

**Conclusion:**

Well-integrated community care requires interprofessional education, collaborative practice, and the participation of community residents in health education.

## Introduction

In response to the rapid increase in diabetes mellitus type 2 (T2DM) patients in Japan, the prevention of onset, early detection and treatment, and promotions aimed at preventing complications among diabetes patients are critical [1,2]. Uonuma City, with a population of about 35,000, faces several issues, such as a declining population, a high ageing rate, and the lowest number of medical doctors per capita in the Niigata Prefecture. The number of medical doctors per 10,000 population is the second lowest [3] and the lowest in Japan when using the physician uneven distribution index [4].

This article describes an experience of treatment and prevention of T2DM, aimed at reducing glycated haemoglobin (HbA1c) to 8% or more, through a campaign named ‘Project 8’, which was started in 2008 at the Koide Hospital and was conducted by an interprofessional team. In 2009, a clinic, a general practitioner, and health and social care professionals participated in the campaign through cross-sector collaboration. Since 2011, the Uonuma School for Community Health and Social Care has implemented a community-based integrated care system [5] based on interprofessional education (IPE) and collaboration in practice (IPCP) and the participation of community residents in health education. Since 2013, the cross-sector collaboration has further expanded, and the hospital has included more professionals in the city for doing rounds in the ward, named the ‘open round’.

The main intervention in the early stage of Project 8 in 2008 was the creation of a Diabetes Mellitus Education and Empowerment Team in the hospital, which focused on treating T2DM. When the Uonuma School was established in 2011, medical students, young trainees, health and social care professionals, and community residents were enrolled. Our sixteen years of experience in the Uonuma area allows us to chronologically describe how each type of integration and intervention was initiated, implemented, and developed.

## Description of the Care Practice

### Screening, Diagnosis, and Treatment in the 1990s and Early 2000s

In 1996, the Niigata Prefectural Medical Association created the Niigata Prefecture Diabetes Screening Study Group to standardise diagnostic criteria for T2DM. The second author (NK), a general practitioner (GP) interested in T2DM, opened his clinic in Uonuma in 1997. In 2006, the Uonuma Area Promotion Council for Diabetes Prevention and Countermeasures was organised, and it has since trained many professionals as certified diabetes educators in Japan [6].

### IPE and IPCP in Uonuma City

In 2008, when the first author (KF) was appointed as the Director of the Niigata Prefectural Koide Hospital, he encountered several advanced diabetic patients. He recorded patients whose glycated haemoglobin (HbA1c) levels were above 8% and were likely to develop complications in the cardiovascular system, eyes, kidneys, or neural tissue [7]. The Diabetes Mellitus Education and Empowerment Team was then established in the Koide Hospital. The team comprised nurses, a pharmacist, a dietitian, a laboratory technician, a physiotherapist (PT)/occupational therapist (OT), a medical social worker, and a home carer in the hospital. The nurses and pharmacist carefully asked the patients about any symptoms of hypoglycaemia due to a possible overdose of an anti-diabetic drug. Nurses in the outpatient clinic contacted patients who did not attend their appointments, reducing non-attendance. The dietitian inquired about their diet, while the PT/OT inquired about their walking and exercise habits.

Monthly team meetings were held to discuss case studies and research issues, such as a ‘survey on the dental health of diabetic patients’, ‘survey on sleep conditions of diabetic patients’, ‘survey on hypoglycaemia’, and ‘survey on drug use’. For example, the team members made ten presentations at the annual meeting of the Japan Diabetes Society (JDS) in 2012. The European Society of Cardiology’s guidelines on diabetes, pre-diabetes, and cardiovascular diseases were introduced in the meetings. The educational hospitalisation programme was standardised and could be efficiently used by multiple professionals within two weeks.

With the transition to proper diabetic drugs and education, the number of advanced diabetes patients decreased after six months. This small success of interprofessional work in the hospital led to a collaboration with a clinic, namely ‘Project 8 (P8)’, which was an interprofessional active intervention for patients [8,9,10]. The second author (NK) was a member of a medical association and promotion committee for diabetes intervention at the time and felt that the success of interprofessional activity was worth extending to the community. At this time, a public health nurse affiliated with Uonuma city, a dentist in open practice, and a health fitness programmer joined the team.

Regarding setting the HbA1c target as ≤8%, the P8 campaign consisted of four components [11]. First, in ‘professional P8’, all medical and healthcare professionals—medical doctors, nurses, pharmacists, dietitians, dentists, and public health nurses—shared their knowledge on treating patients with HbA1c ≥ 8%. Second, in ‘collaboration P8’, patients with HbA1c ≥ 8% at a clinic were referred to the hospital and carefully examined and educated. A clinical path between the hospital and the clinic was developed to share lab data for patient care, involving collaboration between doctors at the hospital and the clinic. Third, in ‘patient P8’, patients and their families were encouraged not to ignore symptoms of HbA1c ≥ 8% or attempt to control their blood sugar level by themselves. Fourth, in ‘checkup P8’, a health checkup facility would refer a person with HbA1c ≥ 8% to the hospital for further examination and treatment if necessary.

During the reorganisation of the medical and social care systems in the Uonuma area, there was a strong push for promoting IPE among health and social care professionals and responding to the needs of community residents to establish a community-based integrated care system [5]. Health and social care professionals, community residents, and the local government must collaborate to ensure the system works efficiently and sustain a high level of health and social care in Uonuma City—which, as noted above, not only has a decreasing population and increasing ageing rate but also has limited resources for health and social care. To educate community residents and improve collaboration between health and social care professionals, the Koide Hospital and the City Medical Association started the ‘Uonuma School for Community Health and Social Care’ in April 2011 [12].

### Establishment of Uonuma School for Community Health and Social Care

#### School curriculum and IPE

The system was named a ‘school’ to reflect its goal of educating professionals and community residents. The school curriculum includes three major themes: ‘learning by medical students and trainees’, ‘learning by community residents’, and ‘learning by health and social care professionals’. Fuse et al. [11] describe the activities of the main school in Uonuma City (population of 35,981 in 2019) [12].

#### Learning by medical students and trainees

The first theme of learning describes how medical students and trainees (young doctors in the first two years of their postgraduate training) became medical professionals of the next generation, learning many types of IPCP in health and social care sites—functioning here as classrooms—not only in hospitals.

Since 2004, the Ojiya-Uonuma City Medical Association has implemented a community medicine training programme. Since 2010, the Department of Community Medicine, designated to Niigata University Graduate School of Medical and Dental Sciences by Niigata Prefecture, has implemented a community medicine training programme for fifth-grade medical students, first as an elective course and later as a two-week compulsory course. Thus, the community medicine training programmes for medical students and clinical trainees were controlled by a programme coordinator in a liaison office of the school. The training programmes at hospitals, clinics, and social care facilities were intricately linked. Consequently, the overall Uonuma area obtained and developed experiences and outcomes as ‘a vital field of medical education in community medicine’. Table 1 shows the number of participants and meetings held from 2011 to 2020. In October 2012, a satellite symposium of ‘All Together Better Health VI’ was held in Uonuma [13].

**Table 1:** Uonuma School for Community Health and Social Care: Cumulative Number of Participants and Various Types of Meetings.


OBJECTS	TYPES OF MEETING	2011–2013	N	2014–2016	N	2017–2020	N	2011–2020	N

	Open school	3,836 a	49	1,439	31	1,413	50	6,688	131

Adult									

residents	Night school	709	22	339	13	97	4	1,145	39

	Class in school	3,872	48	3,530	54	5,325	97	12,727	199

Elementary and junior high school pupils	Open hospital	102	21	166	39	218	52	486	112

Professionals	Interprofessional education and collaboration in practice	3,768	101	3,621	77	4,676	116	12,065	294

Medical students		325		271		490		1,086	

Clinical trainees	From four programmes	86		78		65		229	

	Total medical management			69		179		248	

TOTAL	Cumulative numbers	12,408	241	9,513	214	12,398	319	34,317	774


*Note*: N = number of meetings for residents. a Including 309 participants for ‘All Together Better Health VI’ [13]. Begins in April each year and ends in March of the following year.

#### Learning by community residents

‘Community residents are an indispensable health resource’ is an important catchphrase of the second learning theme. The Uonuma School announces programmes for health promotion classes (lecture-based) at the senior citizens’ club and the community ladies’ club (open school) to which residents can apply. It was efficient to have daytime classes when over 100 residents attended simultaneously. However, interactive learning in classes was also held at the community centre in the evening (night school). The attendees’ understanding was deepened through interactive discussions in smaller groups of 10–20 participants. Community residents will apply to the lectures for self-learning that interests them. Medical doctors, a public health nurse, medical trainees, and students came to a community centre at night, formed ‘kuruma-za’ (literally, sitting in a circle on the floor), and talked with residents. This type of communication is useful as it provides educational content for primary care. T2DM, one of the most common lifestyle diseases, was often discussed (Table 2).

**Table 2:** Numbers of Participants in Diabetes Courses (2011–2020).


OUTCOMES MEETINGS	SUPPRESSION OF INCREASE OF PREVALENCE OF HbA1c ≥ 8%			

	SUPPRESSION OF INDUCTION OF DIALYSIS			

	OPEN SCHOOL NIGHT SCHOOL		RAKU-GOI : COMMON LANGUAGE COURSE	
	
	MEETINGS	PARTICIPANTS	MEETINGS	PARTICIPANTS

Number	97	3,435	17	745

Total	Meetings: 114		Participants: 4,180	

Samples of theme/contents	Prevention of obesity and diabetes Prevention of lifestyle diseases Prevention of hypertension Cigarette and health Community health – present and future Food and health Haemodialysis – Present and future			


Raku-goi: Common language course; i.e., NPG course: Non-professional glossary course.

#### Learning by health and social care professionals

To build the base of the community-based integrated care system, the third theme of learning using IPE and collaboration in practice involves strengthening the network of health and social care professionals(Table 1) [12]. Since 2011, three courses have been offered for learning by health and social care professionals: ‘**Raku-goi**’, ‘**Raku-mon**’, and ‘**Raku-sou**’. The prefix ‘raku’ means ‘freedom’ to remove barriers; it originates from ‘raku-ichi raku-za’, which refers to the free marketplaces and open guilds, a system created by **Oda Nobunaga** (1534–1582), a Japanese great feudal lord. This system was created to unify the country and increase its military and economic power. It removed the previously required taxes, and made it possible for anyone to do business freely [14].

‘**Raku-goi**’ is a common language course where all professionals ‘talking beyond the barrier of professional glossary’ to discuss amongst themselves. These were held 20 times per year. All professionals could attend and talk on various subjects, such as ‘what laboratory tests mean’, ‘what you see with a computed tomography’, ‘activities of the nutritional supportive team’, and ‘care of decubitus’, in the hospital’s lecture hall. Furthermore, professionals from the community explained ‘the work of the comprehensive supportive centre’, ‘what a pharmacy does’, and ‘the community emergency system’ [12].

‘**Raku-mon**’ means ‘Open the gate’, and this course is a programme of interprofessional collaboration in practice. A Koide Hospital ward nurse and clinic GP visited a patient’s home, and along with a care manager.

Since 2013, the hospital has invited a care manager and city drugstore pharmacist to participate in a grand ward round named the ‘open round’. In the open round of the medical department, the medical staff made educational comments and demonstrated practical skills for medical students and other professionals, resulting in the common sharing of medical information in the hospital with visiting staff from home care [12].

The ‘**Raku-sou**’ course is a meeting ‘to remove the barriers in professional thinking’: an interprofessional thinking course. Through Raku-sou, we understood the meaning of professional integration. The course titles have changed every six months since 2012; these included ‘medical ethics of artificially supplying water and nutrition’, ‘comprehensive healthcare systems’, ‘social capital and the aged society’, ‘coaching methods for training new leaders’, and ‘ICF (the International Classification of Functioning, Disability and Health) and the community-based integrated care system’.

Community residents have wished to participate in the health professional course. A Graduate School of the Uonuma School for Community Health and Social Care was established in 2017 to train community residents to become community leaders. The courses in the graduate school have expanded and now cover various topics for multiple professionals [12].

### Reorganisation of the medical care system

The medical, health, and social care systems in the Uonuma secondary medical area were renewed and improved. The Uonuma area (pop. 155,910 in 2020) includes three cities (Uonuma, Minami-Uonuma, and Toukamachi) and two towns (Yuzawa and Tsunan). The Uonuma Kikan Hospital (literally, ‘Nucleus Hospital’) [15] was established in 2015 with 304 beds (increasing to 454 beds in 2021) aimed at providing tertiary care for medical emergencies and advanced medical care, both of which were previously unavailable in the area. The Uonuma City Koide Hospital (formerly prefectural) and three other local hospitals were set up as satellite hospitals. The number of medical doctors—that is, internists, in the Koide Hospital—varied between 2008 and 2018, with five during 2008–2014, four during 2015–2016, three in 2017, and four during 2018–2021. These hospitals and the City Medical Association have attempted to provide the community with the functions of one large hospital. To train medical students and doctors for community medicine, the Niigata University Educational Centre for Community Medicine [15] was also affiliated with the Uonuma Kikan Hospital in 2015.

### Medical ethics

The practice of IPE and IPCP among medical, health and social care professionals and the participation of community residents in health education, implemented by the Uonuma School for Community Health and Social Care, was approved by the Medical Ethics Committee of Koide Hospital.

## Discussion

### Numbers of Participants and Meetings of the Uonuma School

As seen in Table 1, the number of participants and meetings in the Uonuma School during 2014–2016 is less than that during 2011–2013. As the number of hospitals in the Uonuma secondary area was reorganised in 2015, the number of meetings for adult residents from 2015 onwards reduced for open and night schools. Table 2 illustrates the number of meetings and participants specifically for T2DM. As the school started in 2011, cumulative effects from repeated lectures might be a consideration. The number of participants and meetings for elementary and junior high school children did not change from 2011–2013 to 2017–2020.

### Changes in Mean HbA1c and the prevalence of HbA1c ≥ 8.0%

These changes were compared every three to four years from 2005 to 2020. The period 2005–2007 indicates the stage without IPCP. In 2008, the Diabetes Mellitus Education and Empowerment Team was organised in the Koide Hospital. A clinical group comprising nurses (outpatient clinic and wards), a pharmacist, a dietitian, and a laboratory technician was at the frontline to promote and educate the patients and their families, mainly within the hospital.

**Figure 1:**
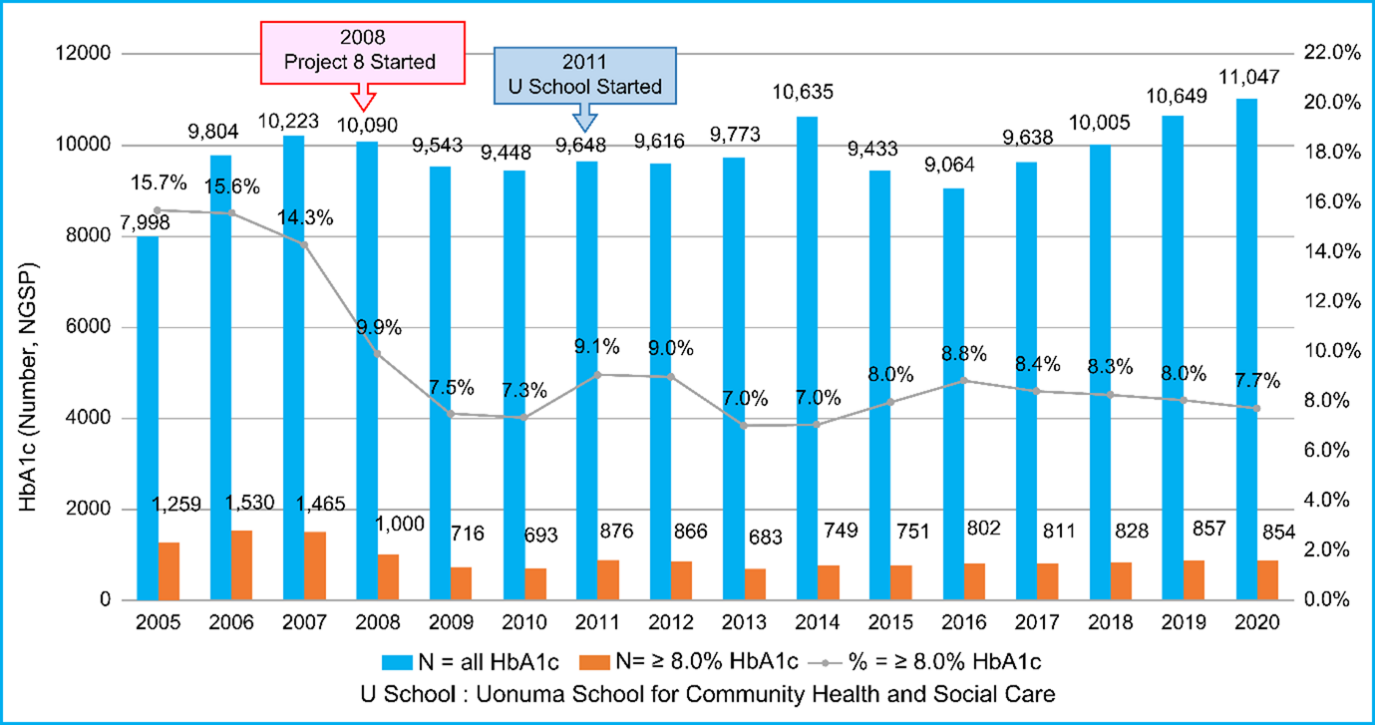
**Comparison of HbA1c values (total and those ≥ 8.0%) before and after the start of Project 8 and the Uonuma School for Community Health and Social Care.** The blue bars depict the total number of HbA1c tests in the Koide Hospital annually from 2005 to 2020. The brown bars depict HbA1c ≥ 8.0% patients. The annual percentages of HbA1c ≥ 8.0% patients for the entire exam are illustrated as a line graph. All values from the Japan Diabetes Society (JDS) units from 2005 to 2011 were converted to National Glycohemoglobin Standardization Program (NGSP) units after 2012. P8 began in 2008, and the Uonuma School for Community Health and Social Care started in 2011.

By the second half of 2008, the rates of HbA1c ≥ 8% patients were decreasing. In 2009, a general practitioner and a health fitness programmer joined; a public health nurse affiliated with a city government and a dentist in the city were also added to the team. The number of HbA1c ≥ 8% patients during 2008–2010 was significantly lower than that during 2005–2007, as illustrated in Figure 1. The period 2008–2010 witnessed the collaboration between a hospital and a clinic and IPCP within the hospital, aiming for HbA1c of less than 8.0%. This period illustrates the importance of general practitioners in the practice of community medicine [16,17]. The period 2011–2013 saw the implementation of IPCP within the hospital, IPCP between a hospital and a clinic, and IPE for community-resident participation following the opening of the Uonuma School. The period 2017–2020 was when the guidelines for T2DM, published in 2017, allowed for the maintenance of slightly higher HbA1c in older people. Figure 3 illustrates the continuous and statistically significant decrease of the prevalence of HbA1c ≥ 8.0% every three to four years from 2011 to 2020, which might indicate the effectiveness of the Uonuma School.

As noted by Fuse et al. [18], as the team had to look for diabetic patients with hypoglycaemic symptoms and perform snow-removal work in winter, the team also had to educate them about risks and the prevention and self-treatment of hypoglycaemia. Since the school’s beginning in 2011, the interprofessional campaign against T2DM has been a key part of the school curriculum (Table 2), and the Uonuma Medical Association (a clinic) has participated in the school programmes.

### Effects of the Reorganisation of Uonuma Hospitals on HbA1c

In June 2015, the Prefectural Koide Hospital, a general hospital (330 beds), was reorganised with the Uonuma City Hospital, a satellite hospital (134 beds). Activities by the new Diabetes Mellitus Education and Empowerment Team, formed under the same Director (the first author) and almost entirely different members, have maintained the same rate of decrease of HbA1c ≥ 8% patients. During 2014–2016, the staff became cognizant of the hospital’s specialisation in managing chronic illnesses in primary care after the reorganisation of hospitals in the Uonuma area in 2015.

At the Uonuma School, topics such as the treatment and prevention of diabetes mellitus have been included in open and night schools every year. The need for IPE corresponds with the necessity of IPCP [19,20]. The P8 campaign thus expanded to the local community [21]. In the Niigata Prefecture and Uonuma City, the respective rates of HbA1c ≥ 8% patients under medical care were 29.7% and 20% in 2008, 27.4% and 16.9% in 2011, 22.7% and 10.4% in 2013, and 19.7% and 8.4% in 2017. Although the rate of HbA1c ≥ 8% patients under the medical care of the prefecture decreased during 2008–2013, that of Uonuma decreased much more. The Uonuma secondary medical area was the only area included in P8 in the 7^th^ Healthcare Programme of Niigata Prefecture circa 2018 [22].

### Various reasons to improve HbA1c

There may also be other reasons for the improvements, such as the activity of a sports club. The Enjoy Sports Club Uonuma, in Koide Town, has been encouraging sporting habits since 2003 across all generations, including older adults [23] and providing sound guidance through enthusiastic general practitioners on the prevention and treatment of diabetes mellitus. Lectures in open and night schools based on interactive learning played an important role in controlling the instances of patients with HbA1c ≥ 8%. HbA1c cases at the Koide Hospital have been significantly decreasing over three-year periods with IPE and IPCP and the active participation of community residents. Thus, interprofessional learning, including the participation of residents, has gradually spread from the hospital and clinics to the community with the support of the Uonuma City government.

**Figure 2:**
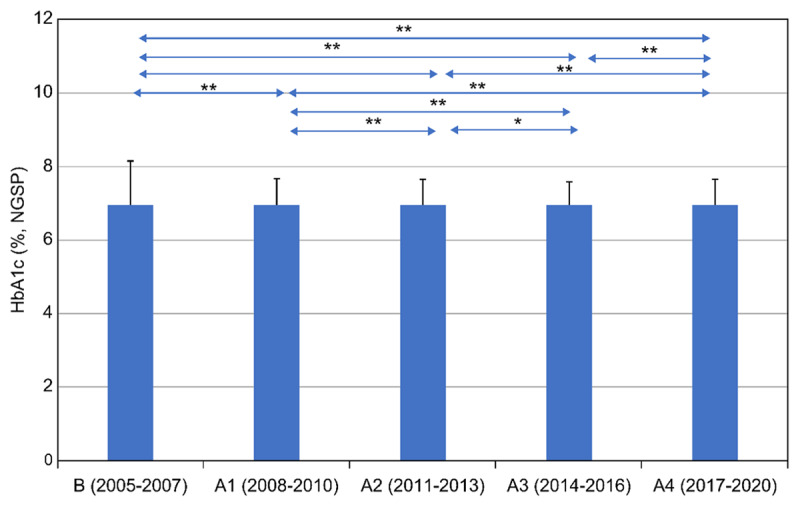
**Comparison of mean HbA1c every three to four years before and after the start of Project 8 and the Uonuma School for Community Health and Social Care.** The X-axis denotes the stage duration (three to four years); the Y-axis denotes HbA1c (%, NGSP). Mean HbA1c every three to four years during 2005–2020 were compared. Stage B, from 2005 to 2007, indicates a period without IPCP. Stage A1, from 2008 to 2010, designates the period of IPCP within the hospital, as well as IPCP between a clinic and a hospital, aiming for HbA1c of less than 8.0%. Stage A2, from 2011 to 2013, shows the period of IPCP within the hospital, and between a clinic and a hospital, and community-resident participation, following commencement of the Uonuma School. Stage A3, from 2014 to 2016, refers to the period when staff became cognizant of the hospital’s specialisation in the management of chronic illnesses in primary care after the reorganisation of hospitals in the Uonuma area in 2015. Stage A4, from 2017 to 2020, is the period when guidelines for diabetes mellitus published in 2017 allowed for the maintenance of slightly higher HbA1c in older adults. A one-way ANOVA test, post hoc test, and t-test with Bonferroni correction were applied to compare all of the integrated criteria. Statistical significance was set as p < 0.05 and p < 0.01. R version 3.6.2 (The R Foundation for Statistical Computing, Austria) was used for all statistical analyses.

**Figure 3:**
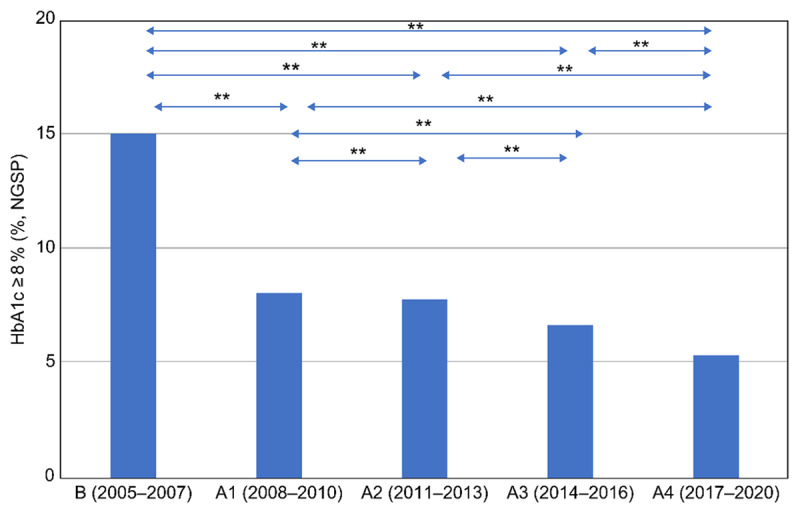
**Comparison of HbA1c ≥ 8.0% before and after the start of Project 8 and the Uonuma School for Community Health and Social Care.** The HbA1c ≥ 8.0% group was compared every three to four years, from 2005 to 2020. The classification of stages from 2005 to 2020 is the same as in Figure 2. A chi-square test, post hoc test, and Ryan’s multiple comparison were applied to compare all integrated criteria. * p < 0.05 and ** p < 0.01 were considered statistically significant. R version 3.6.2 (The R Foundation for Statistical Computing, Austria) was used for all statistical analyses.

In 2017, a combined committee of the Japan Geriatrics Society and the JDS created the Clinical Practical Guideline for the Treatment of Diabetes in the Elderly [24]. The guideline stipulated that individual HbA1c goals of older patients had to be re-evaluated, considering cognitive function, daily living activity, complications, and risk of hypoglycaemia. Despite the guideline allowing individual HbA1c goals to be ≤8.5% [24], the rates of HbA1c ≥ 8% patients in the hospital did not increase (Figures 2 and 3).

### 2020–2022: The COVID-19 Pandemic in Uonuma and Practitioners in Community Medicine

The prevention of infectious diseases has been discussed at the Uonuma School. Although the coronavirus disease (COVID-19) spread in Japan in 2020, the first case in the hospital was only reported on 23 April 2021, when a hospital staff member tested positive on a polymerase chain reaction test for COVID-19. The disease continued to spread, resulting in 38 cases (27 patients and 11 staff, 9 of whom were vaccinated once and 2 of whom were unvaccinated). Patients were treated in the hospital and then transferred to other hospitals, hotels, or homes, and the Uonuma City healthcare centre and the hospital worked to manage the infection. The outpatient clinic was closed for 45 days. No deaths were reported, and the outpatient clinic reopened on 8 June 2021.

### Lessons Learned

#### Participation of community residents and meetings in the Uonuma School

Through the practice/implementation in the last sixteen years in Uonuma city, the main learnings were as follows: first, IPCP among medical, health and social care professionals has been most effective in treating and preventing T2DM. Second, health education is important for not only the patients and community residents but also health and social care professionals. Third, the P8 campaign used the reduction of patients with HbA1c above 8% as a quality indicator to show glycaemic control as a target value when sharing the importance of treatment and prevention of T2DM among patients and their families, health and social care professionals, health checkup facilities, and local governments.

#### Single-professional practice vs. IPCP in hospital or outpatient settings

Various single healthcare professionals have implemented interventions to improve blood glucose levels among patients with T2DM. For example, Subramanian et al. [25] reported the effectiveness of nurse-led interventions on self-management, self-efficacy, and blood glucose levels among patients with T2DM attending a diabetic outpatient department in a hospital in Chennai. Their study concluded that the nurse-led intervention through video-assisted teaching is an effective method for recovering self-management and self-efficacy and reducing fasting and postprandial blood sugar among patients with T2DM [25]. Benedict et al. [26] reported that T2DM therapy management by clinical pharmacists was associated with a greater percentage of patients achieving the Healthcare Effectiveness Data and Information Set goal of HbA1c < 8.0%, reaching the HbA1c goal sooner, and a greater HbA1c reduction from baseline at 3 and 6 months of follow-up compared with patients receiving usual care [26].

Moreover, structured nutrition therapy alone improves glycaemia in comparison to individualised eating plans in overweight and obese patients with T2DM. It also reduces other important cardiovascular disease risk factors like body fat percentage and waist circumference [27]. Kumar et al. [28] also demonstrated the effectiveness of a structured exercise intervention program for insulin resistance in T2DM with a moderate level 2 of evidence. Szafran et al. [29] also observed the significance of IPCP in diabetes care. In Canada, most patients with T2DM are cared for in the primary care setting of family physician practices. Various practice models are used, ranging from a single practitioner to interprofessional team models of care. Physicians affiliated with a primary care network perceived that interprofessional collaboration enabled them to delegate diabetes education and monitoring and adjustment of medications to other health professionals, which resulted in improved patient care [29].

#### Health education for professionals, patients, and community residents

Butayeva et al. [30] evaluated the impact of health literacy, focusing on the impact of health education and interventions on glycaemic control and self-management outcomes on T2DM. The findings demonstrated that health literacy–driven interventions positively impacted glycaemic control and improved self-management behaviours. Glycaemic control and self-management skills improved through individual and telephone-based interventions, respectively.

#### HbA1c as a quality indicator to show glycaemic control

Konnyu et al has reported HbA1c is the commonest reported outcome in quality improvement strategies for adults diabetic patients [31]. At St. Luke’s International Hospital, one of the best hospitals in Japan and where urban residents are under the care of endocrine specialists [32], HbA1c has been advocated as a ‘quality indicator’ in treating diabetes mellitus since 2007. Further, in 2020, the rate of patients older than 65 years of age with HbA1c ≥ 8% was 12.5% at the hospital [33]. At Koide Hospital, the percentage of HbA1c ≥ 8% patients older than 65 years was 7.4% (600/8122 patients) in 2020.

#### What is unique about the achievements in Koide Hospital and Uonuma City?

As seen in Figure 1, the percentage of patients with HbA1c ≥ 8% at Koide Hospital in Uonuma City demonstrated a reduction over a 16-year period from 15.7% in 2005 to 7.7% in 2020.

This is the result of integrated care, aimed at reducing HbA1c to 8% or more based on IPCP, which started in Koide Hospital in 2008 and soon spread as cross-sector collaborative practice with a clinic in the city in 2009; it further expanded to pharmacies and social care facilities in the city through the open round in 2013.

As seen in Figure 2, the periods 2014–2016 and 2017–2020 had fewer participants and meetings than the period 2011–2013. During 2005–2007, the percentage of patients with HbA1c ≥ 8% ranged between 14.3% and 15.7%. After 2008, when the P8 campaign started, IPCP was emphasised, and the percentage of patients with HbA1c ≥ 8% decreased, ranging between 7.3% and 9.9% during 2008–2010. The primary intervention in the Koide Hospital and Uonuma city was the P8 campaign, which began in 2009 when a Diabetic Mellitus Education and Empowerment Team was created in the hospital. The Uonuma School was established in 2011 to educate three groups: medical students and trainees, health and social care professionals, and community residents. Participation in both the open (daytime) and night schools is voluntary. Since 2011, the percentage of HbA1c ≥ 8% patients has been stable between 7% and 9.1% at the Koide Hospital from 2011 to 2020. This demonstrates that both IPE and IPCP among health professionals—that is, health promotion and education for the local community, which might have cumulative effects among community residents—were effective.

The trend of decrease of HbA1c ≥ 8% patients was not only in the Koide Hospital in Uonuma City but also in Uonuma secondary medical area (three cities and two towns) and Niigata Prefecture, which recorded the lowest rate in 2013, with the help of ‘checkup P8’ [34]. Furthermore, since 2007, the incidence in the Uonuma secondary medical area (14 hospitals over three months in 2016, from 1 July to 30 September) was 10.2% (1,237/12,094 patients) [35], and in 2020 (11 hospitals over the same period), it was 7.2% (1,002/13,851 patients). Thus, the reduction in glycaemic control has spread from Uonuma city to Uonuma secondary medical area.

#### Prevalent rate and new induction of dialysis in Niigata and Japan

In 2020, 347,671 patients were treated with dialysis in Japan (out of a total population of 126.3 million) [36]. The number of patients receiving dialysis differed among the 47 prefectures and 344 secondary medical areas. The prefecture with the highest rates of use of dialysis to treat patients in 2020 (prevalent rate) was Tokushima with 398.6; Fukui had the lowest at 225.8, Niigata had the fourth lowest at 236.6, and the national average was 275.4 (adjusted per 0.1 million population). In 2020, the number of newly inducted patients in Japan was 40,744 [37], and the rate of new induction of dialysis adjusted per 0.1 million population was 32.3 in 2020 [36].

T2DM is becoming a major cause of induction for dialysis. In the Niigata Prefecture (population 2,176,879 in 2021), the number of patients receiving dialysis was 5,132 in 2020, and the rate of new dialysis induction per 0.1 million (proportion due to diabetes) was 28.0 (40.8%) in 2020 [38]. In the Uonuma secondary medical area (population 155,910 in 2020), the rate was 7.1 (45.5%) in 2020 [39]. Although age has not been adjusted, there are fewer dialysis patients in the Uonuma area compared with the national average, and the number continues to decrease yearly. Subsequently, the P8 campaign might effectively decrease the number of dialysis inductions in the Uonuma area, including Uonuma City, by successfully maintaining a level of HbA1c of less than 8.0% in the community.

#### National campaign to decrease diabetic nephropathy

Tsuge et al. [40] demonstrated that the 2015 version of this prevention programme targeting the progression of diabetic nephropathy was effective. The revised programme, the 2019 version, was implemented by 146 local governments, and the Ministry of Health, Labour and Welfare advocated for the 2019 version to be used as collaborative practice for preventing the progression of diabetic nephropathy [41]. It was also presented as a campaign manual for local governments, describing the programme in four steps [42]. Although dialysis induction due to diabetic nephropathy has been decreasing (but still accounting for 40.7% of the cause), dialysis due to nephrosclerosis is increasing and was 17.5% in 2020 [36,43].

#### A common goal shared by professionals, community residents, and the local government in Uonuma

The campaign by Uonuma City differs from that of other local governments in terms of who is the initiator and who are the collaborators or followers. With strong leadership, the doctors at the community hospital and a clinic in Uonuma City initiated the P8 campaign in 2008 to monitor the number of people with HbA1c ≥ 8.0% and formed a multi-professional team, with interprofessional education and collaboration in practice, aiming at a common goal. The school implemented continuous, dynamic, and timely lectures in health education for the community residents, with the local government’s involvement. The impact of the Uonuma School has spread from Uonuma City to the Uonuma secondary medical area and the Niigata Prefecture. Thus, the P8 campaign in Uonuma City may be considered an example of a best practice framework in disease management, which started nearly ten years before Japan’s national campaign.

### The Impact could be Improved in Future

Digital innovation and individualised care for preventing and treating diabetes are strongly expected in the future. In 2003, Hirai [44] advocated improving diabetes treatment using a community e-information network (Wakashio Medical Network), making collaborations possible among hospitals, clinics, pharmacies, and nursing departments.

In Hawaii, a community-centric diabetes program will enhance the understanding of diabetes aetiology, diabetes self-management activities, and communication skills that integrated friend-and-family support, community health services, telehealth-enabled diabetes self-management education, and mobile technologies by activating the community’s social capital to support the program for effective disease management support in under-resourced rural communities [45].

For the Uonuma secondary medical area, the regional Integrated Healthcare and Welfare System, ‘Uonuma Mynet’, facilitates the sharing of laboratory data and drug prescription records across hospitals, clinics, pharmacies, and care and welfare facilities in three cities and two towns. This could promote efficient management of T2DM in the area [46].

During 2020–2022, when face-to-face meetings at the Uonuma School were limited due to the COVID-19 pandemic, attendance increased and approximately half participated in online daytime classes. Digital technologies may provide more information and opportunities to improve patient experience and outcomes more efficiently in the future. The JGS/JDS Clinical Practice Guideline for the Treatment of Diabetes in the Elderly (2017) has an individualized treatment goal of HbA1c values between 7.0–8.5% with consideration of their degree of cognition, ability to perform activities of daily living, medication and risk of hypoglycaemia, and age. As a result, treatment and care of T2DM patients are expected to be more individualized in real-time with the help of digital technologies in the future.

The Uonuma School for Community Health and Social Care could provide a platform for digital innovation in community health or broaden its scope to share experiences regarding overcoming various issues of the aged society, reducing the burden on doctors, professionals, and patients with diabetes, similar to the National Health Service Diabetes Programme in the UK [47].

### Limitations

Despite its strengths, this study has several limitations. First, various medications were not included, considering the changing availability and usage of medication over the sixteen years of the study. Second, The Uonuma School for Community Health and Social Care provides self-learning courses for community residents who are highly motivated to improve their health. Although those with low motivation were not included, repeated lectures and various courses over the years would increase motivation, allowing more residents to participate. Lastly, although sporting habits in all generations were encouraged in the Uonuma area, exercise, which is important for preventing and treating T2DM, was not evaluated in the study.

## Conclusions

Uonuma City has a higher percentage of older residents than the rest of Japan. As older people generally have various chronic lifestyle diseases, such as T2DM, Uonuma City faces the challenge of a dearth of resources for medical and welfare services relative to the number of older people in the city. However, we believe that with interprofessional education, collaboration in practice, and the participation of community residents, we could prevent the occurrence and progression of T2DM not only in an individual patient but also in the community as a whole. This campaign contributes towards establishing a sustainable community-based integrated care system with a common goal among residents, professionals, and the local government, hoping to share similar experiences in other rural and urban communities worldwide.
